# Special issue on analysis and mining of social media data

**DOI:** 10.7717/peerj-cs.1909

**Published:** 2024-02-29

**Authors:** Arkaitz Zubiaga, Paolo Rosso

**Affiliations:** 1 School of Electronic Engineering and Computer Science, Queen Mary University of London, London, United Kingdom; 2 Technical University of Valencia, Valencia, Spain

**Keywords:** Social media, Data mining, Natural language processing, Computational social science

## Abstract

This Editorial introduces the PeerJ Computer Science Special Issue on Analysis and Mining of Social Media Data. The special issue called for submissions with a primary focus on the use of social media data, for a variety of fields including natural language processing, computational social science, data mining, information retrieval and recommender systems. Of the 48 abstract submissions that were deemed within the scope of the special issue and were invited to submit a full article, 17 were ultimately accepted. These included a diverse set of articles covering, *inter alia*, sentiment analysis, detection and mitigation of online harms, analytical studies focused on societal issues and analysis of images surrounding news. The articles primarily use Twitter, Facebook and Reddit as data sources; English, Arabic, Italian, Russian, Indonesian and Javanese as languages; and over a third of the articles revolve around COVID-19 as the main topic of study. This article discusses the motivation for launching such a special issue and provides an overview of the articles published in the issue.

## Introduction

The number of users in online social media has increased substantially in the last two decades. With the growing number of users posting and resharing content, interacting with others and exchanging files, online social media platforms have become an invaluable data source to perform scientific analyses at scale. These analyses are conducted in a wide range of scientific fields, including research in computational social science, understanding social phenomena, natural language processing at scale and social network analysis investigating how interactions are shaped online, *inter alia*.

Along with the many advantages that social media platforms have offered with unprecedented means for communication with one another, they are also often misused for malicious purposes. Indeed, there is evidence of many users exploiting social media platforms to perform uncivil actions and engage in toxic conversations. This can cause harmful consequences for online users, many of whom have no other option but to delete their social media accounts. Detection and mitigation of this kind of activity has become a priority in the scientific community, as is the need to further analyse, measure and understand this phenomenon.

With these and other related challenges in mind, this special issue sought a diverse set of contributions with novel research analysing and/or mining social media data to address a wide range of priorities in the scientific community, including but not limited to: (i) analysis; detection; resolution and mitigation of online malicious communications, (ii) computational social science; social network analysis and social computing, (iii) measurement; analysis and modeling of social media–including real-time analytics, (iv) data collection and annotation from social media, (v) trust, privacy and security in social media, (vi) natural language processing for social media, (vii) recommender systems for social media, and (viii) information retrieval and search in social media. Topics beyond those listed above were also welcome as long as the fit to the scope of the special issue was clear.

## Special Issue Themes

The special issue received a total of 74 abstract submissions, of which 48 were deemed within the scope of the issue and were invited to submit full articles for review. After peer review, 17 articles were ultimately accepted to be included in the special issue.

Among the published articles, we identify a diverse range of themes which make up the special issue. As many as six articles focus on **sentiment analysis** for different purposes (Smetanin, 2022; Pratama & Firmansyah, 2022; Baxi, Philip & Mago, 2022; Nguyen & Gokhale, 2022; Shamoi et al., 2022; Ali, Irfan & Lashari, 2023). Four studies focused on tackling **online harms** of different kinds, with studies on abusive language detection (Almerekhi, Kwak & Jansen, 2022; Ramponi et al., 2022), suicidal ideation detection (Baghdadi et al., 2022) and misinformation detection (Obeidat et al., 2022). Others studied **NLP techniques for social media**, focused on the analysis of Twitter discourse (Heaton et al., 2023), language identification (Hidayatullah et al., 2023) and named entity recognition (Fudholi et al., 2023). There are also a number of **analytical studies** that investigate different societal issues, including gender equality through advertising data (Al Tamime & Weber, 2022), analysis of gender-based violence across countries (Rimjhim & Dandapat, 2022) as well as electoral data (Yang, Hui & Menczer, 2022). Another study looked at the **effect of visual images** on social media news (Al-nuwaiser, 2022).

All in all, these articles make a diverse contribution to the literature of analysing and mining social media data, highlighting the wide range of research questions that social media as a data source enables us to answer.

Further, looking at the diversity in the published articles, we look at three different dimensions:

• **COVID-19:** given the temporal overlap of the special issue with the pandemic, it attracted six submissions related to COVID-19.
• **Social media platforms:** by far the most common social media platform used as a data source was Twitter, with 13 articles. Two articles use Facebook data, one uses Reddit data and one uses web data.
• **Languages:** while most studies focus on English data collected from social media, a few of the articles use data in other languages. Indeed, there are two articles using Arabic data, one using Italian data, one using Russian data and one using Indonesian and Javanese data.

## Summary of Contributions

The 17 articles accepted to the special issue include the following, in order of publication:

1. Al Tamime & Weber (2022) used advertisement data collected from Facebook to analyse the interests of different demographic groups in different types of advertising. They were particularly interested in looking at gender differences across advertising related to science as opposed to other topics. They primarily intended to investigate if the interests (or lack thereof) in science advertising would echo societal biases leading to reduced engagement of women in STEM. While they found a range of differences in demographic groups and interest in different kinds of advertising, their study concluded that there is little evidence suggesting that advertising data can measure the decline in interest in STEM for young women in the USA.
2. Yang, Hui & Menczer (2022) studied the impact of inauthentic user accounts such as bots on distorting the political discourse in social media. They did so by collecting tweets associated with a 2018 US midterm election. They proposed a method to identify voters on Twitter and systematically compare their behaviours with different random samples of accounts. This then enabled their analysis looking at the effects of inauthentic accounts. They found that some accounts do indeed flood the public data stream with political content, leading to an over-representation of the activity of these accounts in datasets and reducing the visibility of the voice of others.
3. Smetanin (2022) provided new resources enabling sentiment analysis in the Russian language, which had been limited to date due to the lack of resources comparable to other languages such as English. The author sampled tweets from the publicly available Twitter Stream Grab to end up with a collection of more than 13K tweets annotated for sentiment by multiple annotators. Tweets were manually categorised into one of five classes: positive, neutral, negative, speech act, and skip. With this study, the author also released a novel transformer-based sentiment classification model for the Russian language.
4. Rimjhim & Dandapat (2022) investigated social media data to understand if it can reveal insights about gender-based violence across different countries. To tackle this, the authors look at correlations between social media content linked to gender-based violence and government statistics across different countries. The authors employ a range of methods to analyse these correlations, including graph-based methods. The study concludes that countries with similar cultures show similarity in social media content about gender-based violence, thus validating the hypothesis that content in social media may reflect societal behaviour when it comes to gender-based violence.
5. Almerekhi, Kwak & Jansen (2022) investigated changes in online behaviour of users who publish in multiple communities on Reddit by measuring their toxicity levels. They first automatically labelled a large collection of over 87 million posts as toxic or non-toxic, which they then analysed. The study aimed to identify toxicity changes by a user within the same community, across multiple communities, and over time. The study revealed, among others, that a user’s toxic behaviour is highly dependent on the toxicity level of the community they participate in, suggesting that users adapt their behaviour to the norm of the communities in question.
6. Baghdadi et al. (2022) created a new Twitter dataset with Arabic tweets, where tweets are labelled as suicidal or not. The objective is to develop a model which can detect suicidal ideation from tweets, as a binary classifier that can identify these problematic cases to act upon early on. After collecting a dataset which was labelled by five different annotators, they experimented with a range of models. Among the models under study, they found that a transformer-based model that used Arabic BERT achieved the best performance. Their study is among the first contributions to study suicidal ideation detection in the Arabic language.
7. Pratama & Firmansyah (2022) investigated the engagement of political leaders and health organisations with their audience in social media during the COVID-19 pandemic. To measure the engagement, they focus on the concept of online societal association. They collected a dataset with over 173K tweets posted by political leaders and health organisations, which they analysed. Among the political leaders, they found that the one that engaged the most was the Primer Minister of the United Arab Emirates, whereas among the health organisations the one that prevailed was the Public Health Agency of Canada. The study highlights the importance of effective communication through social media during pandemics.
8. Baxi, Philip & Mago (2022) examined how the pandemic was reported in the news as well as differences in public reaction to the pandemic in the West and the East. They used archival data from Facebook posts associated with COVID-19 news and posted by English-language mass media between 2020 and 2022, with a total collection of over 700K posts. They use the Valence Aware Dictionary and sEntiment Reasoner (Vader) to measure the news tone and the sentiment polarity score. Their study concludes that posts about the pandemic were more negative in the West than in the East.
9. Nguyen & Gokhale (2022) introduced a classification method to detect anti-government sentiment from tweets. They studied the effectiveness of such classification algorithm during COVID-19 anti-lockdown protests in the USA. The authors collected, annotated and released their own datasets associated with two events, namely Operation Gridlock and anti-lockdown protests in Michigan. By combining a range of text-based and user-based features to perform the classification, the study shows that the proposed method could effectively identify anti-government sentiment in tweets with an accuracy of 85% and an F1 score of 0.82.
10. Ramponi et al. (2022) studied hate speech expressed in social media which is motivated by religious beliefs. The study makes a distinctive contribution to the hate speech detection literature which had largely focused on other kinds of hate such as racism, sexism and misogyny, but had overlooked the problem of religious hate. Authors introduce a novel Twitter dataset containing samples of religious hate in two languages, English and Italian, and perform benchmark classification experiments on these two new datasets. They published the datasets aiming to encourage researchers to conduct further research in this understudied problem.
11. Shamoi et al. (2022) studied the sentiment expressed by social media users towards vegan diets. They collected a Twitter dataset discussing vegan diets and used a method based on mutual information to extract sentiment-related information from tweets and derived their sentiment towards veganism. Their study revealed that veganism is becoming increasingly popular in recent years and is currently framed more positively than in previous years. In addition to increased positivity, however, they also found increasing rates of expressions of fear towards vegan diets, which shows a potential sign of increased polarisation between supporters and opponents of veganism.
12. Obeidat et al. (2022) developed a novel Twitter dataset for misinformation detection from social media, with a specific focus on COVID-19 misinformation. The authors argue that misinformation can occur in many forms and can have different levels of impact on society, and therefore develop a fine-grained annotation schema that includes 19 different types of misinformation. They carefully curated annotation guidelines to enable high-quality annotation within this annotation schema. Following these guidelines, they labelled a dataset with 6.7K tweets which they make publicly available.
13. Al-nuwaiser (2022) conducted a user study through crowdsourcing platforms to investigate the impact of using images with news in social media. They investigated news posted on the Facebook platform, and assessed users’ perceptions of images of three different types: data visualisation (directly about risk information), advisory (not containing direct risk information, but instead help on how to lower risk), or clickbait (containing no risk-related information, just generic visuals). The study highlights the importance of using images with news reporting in social media, showing the importance of choosing the right type of images.
14. Heaton et al. (2023) made a critical reflection on how techniques for computational linguistics are being used to analyse Twitter discourse with different research objectives in mind. They focus on three different techniques for language analysis, which include topic modelling, sentiment analysis and emotion detection. The authors highlight limitations in how these methods are currently being used and how their outputs are being interpreted, suggesting a more careful research design. Among the challenges encountered, they find that the presence of negation and sarcasm in social media posts jeopardises the performance of the aforementioned techniques, which need further exploration.
15. Ali, Irfan & Lashari (2023) performed an analysis of sentiment towards COVID-19 countermeasures taken by the Pakistani government during the years of 2020 and 2021. To enable this analysis, the authors collected datasets from the social media platform Twitter using the Snscrape collection tool, which they subsequently annotated. They implemented and compared four state-of-the-art sentiment classification models, which included Valence Aware Dictionary and sEntiment Reasoner (VADER), TextBlob, Flair, and Bidirectional Encoder Representations from Transformers (BERT). They found that the transformer-based model, BERT, was most accurate among the tested settings, where a fine-tuned BERT model achieved an overall accuracy of 92%.
16. Hidayatullah et al. (2023) focused their research on the language identification task. That is, given a text as input, developing a model that automatically identifies the language used in the input text. Their primary focus is on code-mixed language in the cases of the Indonesian, Javanese and English languages. They collect and annotate a Twitter dataset to conduct experiments on language identification. Through testing a range of different state-of-the-art classification models, they find that a fine-tuned IndoBERTweet model performed best.
17. Fudholi et al. (2023) suggested the development of a named entity recognition system to facilitate the analysis of tourism-related information from social media. Indeed, information can be overwhelming in social media if not conveniently organised, and therefore named entity recognition could help organise comments by location, for example. To tackle the problem, they developed a BERT-based named entity recognition system, which achieved competitive performance with an F1 score of 0.80. They also conducted a survey to quantify user satisfaction, which was overall above a score of 4 in a scale from 1 to 5.

## Concluding Remarks

The special issue attracted a large number of submissions, of which 17 were ultimately accepted to be included in the issue. These articles include a wide range of topics within the umbrella of analysing and mining social media data. While there is some diversity in terms of social media platforms and languages studied, we believe that these are among the key dimensions that would need expanding in future research.

When it comes to languages, there is an urgent need to analyse, and develop methods for, data written in different languages. This is crucial to enable generalisability of the methods and insights, and to make sure that our analyses cover all regions of the world, including under-represented areas.

Looking at the social media platforms studied, Twitter has been, until 2023, predominantly used as a data source for social media studies. This has been the case primarily because of its generous access to a free API, which provided access to large datasets. As this has recently changed, where Twitter charges a fee for access to its API, which is not affordable for the majority of the scientific community, there is an urgent need to move on and find alternative platforms to research.

## Additional Information and Declarations

### Data Availability

The following information was supplied regarding data availability: This is an Editorial.
